# Behavioral, Normative and Control Beliefs about Earthquake Preparedness: A Deductive Content Analysis Study

**DOI:** 10.1371/currents.dis.20fbad29d53fb164ac2699dc2736d804

**Published:** 2018-09-28

**Authors:** Mehdi Najafi, Hamid Reza Khankeh, Helen Elmi, Negar Pourvakhshoori

**Affiliations:** Department of Health in Emergency and Disaster, University of Social Welfare & Rehabilitation Sciences, Tehran, Iran; Red Crescent Society, Iran; Department of Health in Emergency and Disaster, University of Social Welfare and Rehabilitation Sciences, Tehran, Iran; Department of Clinical Science and education, Karolinska Institute, Stockholm, Sweden; Consultant Psycholgist, Azad University, Tehran, Iran; Post-doc fellowship in Climate Change, Health in Emergency & Disaster Research Center, University of Social Welfare & Rehabilitation Sciences, Tehran, Iran

## Abstract

Introduction: Despite efforts to increase earthquake preparedness (EP), the level of earthquake preparedness in Tehran is low, even when people acknowledge the risk they face. This problem has its roots in the beliefs of Tehran inhabitants about EP. This study is aimed to elicit the salient beliefs about earthquake preparedness among Tehran citizens.

Method: This is a deductive content analysis research. The theory of planned behavior (TPB) has been applied as the theoretical framework of the study. 132 semi-structured interviews have been conducted with Tehran heads of households and the obtained data have been analyzed.

Results: The interviews showed that the belief in the usefulness of the EP and the belief that “the EP can cause anxiety among family members” were the salient behavioral beliefs (the ones influencing the attitude towards a behavior). The main normative belief (which influences the subjective norms), was “my family doesn’t disagree with the EP” although most of the interviewees did not know about their families’ views. Finally, the main control belief (which is the basis of perceived behavioral control), was that “education can facitilates EP”.

Conclusion: Tehran inhabitants preparedness behaviors can be influenced by their behavioral, normative and control beliefs about preparedness. Recognition of these beliefs may assist policy makers and executives to develop a better understanding of the origins of the preparedness behaviors. Any interventions to change these behaviors should be made based on such knowledge.

Key words: Earthquake; preparedness; salient beliefs; theory of planned behavior

## **Introduction**

Tehran, the capital of Iran, is located at the foot of the Alborz Mountains Range which is a part of the Alpine-Himalayan Orogenic Belt. The city has great seismic potential with its numerous active faults. Tehran has not experienced a severe earthquake in the past 150 years, but a number of massive earthquakes has been recorded in its history^ 1^^,^ 2^,^ 3. Apart from its geological position, Tehran’s rapid population growth of over 12 million people has made it more vulnerable to earthquakes^4^. According to the studies, Tehran’s probable earthquake might result in serious losses and damages^5^. Nevertheless, earthquake preparedness of Tehran inhabitants is extremely low^6^. Of course, this is not the case merely in Tehran. The earthquake preparedness level in some of the cities prone to earthquake in some countries also is low^7^^,^ 8 while more or less the people are aware of the risk. Thus, people’s knowledge of the risk does not necessarily result in the people’s preparedness^9^.

According to the studies conducted on disaster preparedness, several factors can affect the preparedness including risk perception^10^^,^ 11^,^ 12^,^ 13 preparedness perception^14^^,^ 15^,^ 16 critical awareness^12^^,^ 17^,^ 18 optimistic and normalization biases^19^^,^ 20 self-efficacy^14^^,^ 21^,^ 22^,^ 23 collective efficacy^24^ fatalism^13^^,^ 22^,^ 25^,^ 26^,^ 27 locus of control^13^^,^ 23^,^ 27 anxiety^7^^,^ 12^,^ 27 previous disaster experience^11^^,^ 13^,^ 28^,^ 29 societal norms^30^ sense of community^31^ community participation and empowerment^32^^,^ 33 social trust^34^ perceived responsibility^11^^,^ 15 responsibility towards others^18^ coping style^14^^,^ 21^,^ 35^,^ 36 and available resources^32^^,^ 37.

Few studies have tried to identify the capability of people’s beliefs in influencing the earthquake preparedness behaviors. The main problem in most of these studies is a lack of theoretical underpinning. Since applying a theory to develop evidence-based knowledge is a requirement, this study makes an effort to apply a suitable theory to identify the people’s beliefs which may influence the preparedness behaviors. Understanding of belief-preparedness relationships can facilitate the development of preparedness behaviors in community members. To explain the earthquake preparedness behaviors, various theories and models can be employed including: Theory of Planned Behavior (TPB)^38^^,^ 39^,^ 40^,^ 41 Protection Motivation Theory (PMT)^16^^,^ 42 Person Relative to Event Theory (PrE)^21^^,^ 43^,^ 44^,^ 45^,^ 46 Protective Action Decision Model (PADM)^47^^,^ 48 and Social-Cognitive Preparation Model^12^ (Here, only the most used relevant models and theories are cited. There are other models and theories in the social psychology domain that are not cited here). Among these, the theory of planned behavior (TPB), as far as it is concerned, has not been applied to explain the earthquake preparedness behaviors^49^. The TPB is a belief-based theory. As we wanted to find the beliefs concerning earthquake preparedness behaviors, the TPB has been applied as the theoretical framework of the study. The aim of this study was to elicit the salient beliefs relevant to earthquake preparedness among Tehran inhabitants.

According to Ajzen, who has theorized TPB, “human behavior is guided by three kinds of considerations: beliefs about the likely outcomes of the behavior and the evaluations of these outcomes (behavioral beliefs), beliefs about the normative expectations of others and motivation to comply with these expectations (normative beliefs), and beliefs about the presence of factors that may facilitate or impede performance of the behavior and the perceived power of these factors (control beliefs). In their respective aggregates, behavioral beliefs produce a favorable or unfavorable attitude toward the behavior; normative beliefs result in perceived social pressure or subjective norm; and control beliefs give rise to perceived behavioral control. In combination, attitude toward the behavior, subjective norm, and perception of behavioral control lead to the formation of a behavioral intention. As a general rule, the more favorable the attitude and subjective norm, and the greater the perceived control, the stronger should be the person’s intention to perform the behavior in question. Finally, given a sufficient degree of actual control over the behavior, people are expected to carry out their intentions when the opportunity arises”^50^. Figure 1 schematically illustrates the theory of planned behavior^51^. In order to properly plan for disaster situations, it is vital for policymakers and emergency responders to understand the attitudes, concerns, and reactions of individuals and families caught in a disaster. Moreover, this information can guide the development of disaster preparedness educational materials or programs for the general public^52^. TPB can provide a framework for these activities.

**The theory of planned behaviour.**
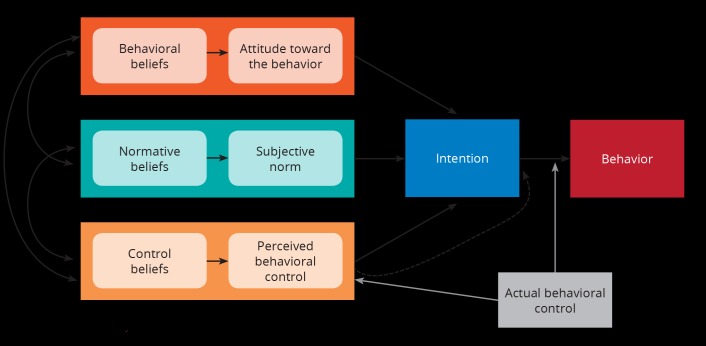

## **Methods**

This is a deductive content analysis research. The deductive approach is based on previously theoretically derived categories and the initial coding starts with a theory. Based on this theory, the researchers embark on identifying the key concepts as the initial coding categories of the analysis, bringing them in connection with the text^53^. This explorative study is meant to elicit the commonly held beliefs and identify the content of behavioral, normative and control beliefs that are shared by the target population about earthquake preparedness based on the TPB. The data have been collected through interviews with Tehran citizens.

**Sampling**

Tehran consists of 22 districts and 134 sub-districts. 132 households of different socioeconomic backgrounds were selected from 22 districts of the city. First, three sub-districts were selected randomly in each district. Second, the two households were purposefully selected following a 'maximum variation sampling' approach within each selected sub-district. Finally, the heads of the households were selected as respondents, since they are considered to be the main decision makers. More specifically, the research team contacted the sub-districts council to support this study. The members working in the respective sub-districts council were asked as key informants to select the households in each socioeconomic group. The requirement for selecting the participating households was that they had stayed in Tehran for at least 10 years. Table 1 shows the participants’ characteristics.

**Table 1. Participants’ Characteristics (N=132):** 

	Percent	Mean	SD
Male	83		
Age		44.34	12.41
Married	87.9		
Years of education		12.5	3.7
Employed	75		
Years of living in Tehran		25.7	5.2
Had home ownership	51		
Families with more than two members	86.4		
Had disaster experience	25		
Prepared (DPBscore ≥ 5)	10.2		

**Materials**

Since the TPB is especially applied to behaviors, the behavior elements of the Public Readiness Index (PRI)^54^were adopted which is scored on a scale from 0 to 7, for defining the earthquake preparedness behavior (Table 2).

**Table 2. The behavior elements of the Public Readiness Index (PRI):** 

Question	
1	Have you actually prepared a disaster supply kit with emergency supplies like water, food and medicine that is kept in a designated place in your home?
2	Have you actually prepared a small kit with emergency supplies that you keep at home, in your car or where you work to take with you if you had to leave quickly?
3	Have you actually made a specific plan for how you and your family would communicate in an emergency situation if you were separated?
4	Have you actually established a specific meeting place to reunite in the event you and your family cannot return home or are evacuated?
5	Have you actually practiced or drilled on what to do in an emergency at home?
6	Have you actually volunteered to help prepare for or respond to a major emergency?
7	Have you actually taken first aid training such as CPR in the past five years?

Moreover, a semi-structured interview approach was adopted to explore the participants’ beliefs about the earthquake preparedness. Although the interviewees could freely talk about their beliefs on the earthquake preparedness, a set of questions based on the TPB had to be answered too^55^ . If needed, the participants were consulted about the questions. The questions were already designed by an expert panel (Table 3).

**Table 3. Interview Questions:** 

Question
What do you think about the advantages of earthquake preparedness?
What do you think about the disadvantages of earthquake preparedness?
Is there anything else you would like to say about the advantages and disadvantages of earthquake preparedness?
What does your family think about earthquake preparedness?
What do your friends think about earthquake preparedness?
Is there anything else you would like to say about other persons’ ideas regarding earthquake preparedness?
What factors or circumstances can contribute to your earthquake preparedness?
What factors or circumstances make the earthquake preparedness difficult or impossible for you?
Are there any other issues striking to your mind regarding the earthquake preparedness?

**Procedure**

The study was conducted from January to February 2015. Two preliminary interviews were conducted to test the quality of the questions. The interview guideline was developed based on previous studies^56^ and expert opinions. The participants were interviewed by trained interviewers in their homes. They were asked whether they would participate in a study on “peoples’ attitudes towards earthquake preparedness”. Written consents were received from the participants and no identifying data was collected. All interviews were conducted in persian language. They were recorded electronically and transcribed verbatim. Each interview took 30 minutes in average. The study was approved by the Research Ethics Committee of Tehran University of Medical Sciences.

**Analysis**

A deductive content analysis was used to analyze the data. First, a structured categorization matrix based on the TPB was developed. Then, all the data were reviewed for content and coded for correspondence with the identified categories^57^. To increase the validity of the analysis, the data coding was done by two researchers independently. The data were analyzed based on main concepts which have been extracted from the theory.The data collection continued until the study reached saturation, in which no new information was provided. The MAXQDA software was used to process, order and compare the codes.

## Results

The data extracted from the interviews were divided into three main categories: behavioral beliefs, normative beliefs and control beliefs. Behavioral beliefs included the advantages and disadvantages of the expected behaviors in earthquake preparedness. Normative beliefs were related to the approval or disapproval of the behaviors by the people whose views are important for the interviewees. Control beliefs were facilitating factors or barriers of the earthquake preparedness behavior from the interviewees’ point of view. The content of behavioral, normative and control beliefs was identified from the transcripts by two researchers who were familiar with TPB (Table 4).

**Table 4. Codes of behavioral, normative and control beliefs that extracted from the data:** 

	Codes	Quotation Count
Behavioral beliefs	Preparedness reduces casualties and losses of my family	80
	Preparedness causes anxiety in my family	55
	Preparedness makes my family members aware of what to do in an earthquake	40
	Preparedness is useless	30
	Preparedness protects my family as long as the relief workers have not arrived.	20
	Preparedness helps my family members find each other after the disaster	20
	Preparedness helps my family prove their identity and their ownership of the properties and assets	15
Normative beliefs	My family doesn't disagree with the earthquake preparedness	55
	My family approves the earthquake preparedness	40
	My friends approve the earthquake preparedness	30
	Some of my friends approve and some of them disapprove the earthquake preparedness	30
	My family and those around me have no opinion on the earthquake preparedness	20
Control beliefs	Educating makes it easier to prepare my family for the earthquake	122
	Daily involvements are obstacles for preparing my family	55
	Indolence and nonchalance are obstacles for preparing my family	40
	Financial restrictions are obstacles for preparing my family	40
	Earthquake is an act of God and I cannot influence its consequences by earthquake preparedness	20
	Living in the apartment is an obstacle for preparing my family	15
	My family’s preparedness depends on the other families’ preparedness	15
	There is no barrier for preparing my family	10

**Behavioral beliefs**

Although 80.8% of the interviewees were not prepared for earthquakes, they believed that earthquake preparedness is useful. When they were asked to comment on the advantages of earthquakes preparedness, they often pointed out the reduction in casualties and damages. One of the interviewees stated: *“the most important advantage of readiness is that our lives would be less in danger. Besides, if we are prepared, our financial losses will be reduced.”*

Some of the interviewees believed that the earthquake preparedness might minimize the confusion in case of an earthquake. One of them stated: *“if we get prepared, we are aware what to do in an earthquake.”*

The statements such as those mentioned do not directly highlight the ultimate advantage of the earthquake preparedness, but they evidently show that the interviewees expected positive consequences from such preparedness. Some people mentioned some particular issues in this case such as protecting the family until relief workers arrive, finding the family members after the earthquake, the proof of identity, the proof of ownership of properties and assets and the like. On the other hand, for various reasons, some of the interviewees thought that the earthquake preparedness was useless. The most important reason behind such a claim is thinking about the worst possible scenario. One of the interviewees said: *“In the current situation of Tehran, how would preparedness be beneficial? If a strong earthquake occurs, Tehran will be reduced to rubble. I do not think anyone could do anything.”*

Thinking about the worst possible scenario, namely reducing into rubble, was common among almost all of the participants. Even those who believed earthquake preparedness was useful felt doubt about it. One of the interviewees, a professor of geomorphology, admitting the preparedness advantage, stated: *“when our house is ruined, how could we access the tools and facilities prepared? So, it is not clear that the providing of these things would be advantageous.”*

*There were few people who believed that although earthquake preparedness was good, there was no point in preparing for something which might never happen. One of the interviewees stated: “It is so good to get prepared, but why should one get prepared for something which might never happen or for something which causes so extensive damages that it makes preparedness useless.”*

In this statement, the interviewee made reference to two of the widespread beliefs about earthquakes: first, “earthquake might never happen, so preparedness is not necessary", and second: “if a devastating earthquake happens, it will ruin everything including what we have prepared”. In fact, the first belief refers to “no need to the preparedness because of the earthquake uncertainty” and the second one refers to “the preparedness uselessness in the case of the worst possible earthquake scenario”. Believing that “earthquake preparedness is not necessary” is about the preparedness. This is not a behavioral one, since it does not refer to any positive or negative expectations from the earthquake preparedness. Then, “the earthquake preparedness is not useful” can be considered as a behavioral belief. Whereas the main benefit of the earthquake preparedness is considered as reducing the casualties and damages, this belief would mean “the earthquake preparedness does not reduce the casualties and damages”.

Some of the participants believed that the earthquake preparedness would cause anxiety among family members. One of them stated: *“One of its disadvantages is that it provokes anxiety in their family over the earthquake. This can highly affect the living very much and may lead to an excessive stress.”*

This clearly refers to the negative expectation about the preparedness. In other words, this can be categorized under behavioral beliefs. No other case was found in the data concerning the preparedness disadvantages.

**Normative beliefs**

Most of the interviewees didn’t know the ideas of their family and friends about the earthquake preparation. It seems that it was not so important for them to be asked about it. One of them stated:* “I don’t know the views of my family and friends, because we do not talk about these things at all.”*

A lack of knowledge about those whose views are important to the interviewees is a key finding of the current study. However, most of these people thought their family members and friends do not disagree with the earthquake preparedness. Nevertheless, “not disagree” cannot quite be equivalent to “agree”. This normative belief more or less means “the people around me neither agree nor disagree with the preparedness”. Even those claiming that their families approved earthquake preparedness , had not frankly asked about the families’ opinion. It was evident that the interviewees did not feel a sense of pressure on the part of their acquaintances in this regard. One of them pointed out:* “Most of my family members and friends do not even think of it. I can say that almost all of them are indifferent to it.”*

This in fact implies a normative belief which can be formulated as follows: "the people around me have no opinion about the earthquake preparedness". Although this belief does not disapprove the preparedness, therein no point in approving it.

**Control beliefs**

Most of the interviewees believed that “the public education” could help their families for earthquake preparedness. Most of them believed that informing and educating (especially via radio and television) could facilitate the earthquake preparedness. This statement is an equivalent to the control belief that “if I am informed, it would be easier for me to prepare my family for earthquakes”.

Most of the interviewees believed that “daily involvements” did not leave any room to get involved in the earthquake preparedness. One of the participants remarked:* “The peoples are so busy, so they have no time to think about these things.”*

“Indolence and nonchalance" were also believed to be obstacles to preparedness. This belief clearly shows an internal barrier influencing the person’s behavior. One of the interviewees stated: *“I think the main obstacle is indolence and nonchalance not restricted to this matter. An indolent nonchalant person deals with everything in the same way.”*

“Financial constraint” was reported by some of the interviewees as a barrier. They believed that preparedness incurred expenses which might be difficult to bear. One of them remarked:* “preparedness incurs some expenses. With our low incomes, it is not even at the bottom of our to-do list.”*

The other interviewee believed that it was not a family responsibility to pay for the preparedness. He pointed out:* “if the lives of the people are really worthy for the government, it should provide us with preparedness packages for free or with very low prices.”*

This statement means that the government is responsible for the family’s preparedness, because families have extra expenses more critical than the preparedness costs.

Some of the interviewees believed that if it is “destined” to die, it happens, no matter to be prepared or not. One of them remarked: *“if it is destined to die, it happens. Earthquake or the like is a cover. It is not a matter of getting prepared or not.”*

Most people who believed in preparedness also believed in “the destiny”. One of them remarked: *“we should try our best, leave the rest to God. We cannot control everything.”*

The statements like these imply the control belief “earthquake is an act of God and I cannot influence its consequences by the earthquake preparedness”. It seemed that the people with such beliefs regard the main source of control somewhere out of reach.

Some of the participants thought they could not be prepared in the “apartments”. This mental image is rooted in the belief that preparedness kits should be kept in a safe place, while in an apartment completely destroyed in an earthquake, it is not possible. This belief implied again the worst possible scenario. One of the interviewees believed:* “there is no safe place in an apartment for keeping the preparedness kits. If we had a house, we could keep the equipments in the stockroom in the corner of the courtyard. Now in the absence of such a house, we cannot prepare for an earthquake even if we desire.”*

These statements are indicative of the control belief “living in an apartment is an obstacle for preparing my family”.

The beliefs such as “the community readiness leads to my family’s preparedness” and “the whole building preparedness is a necessity for my family preparedness” are equivalent to the control belief “my family’s preparedness depends on the other families’”. Few people believed that there was no barrier for preparing their families.

The interviews clearly showed that there were some salient behavioral, normative and control beliefs about the earthquake preparedness which can lead to the participants' relevant behaviors. Namely, some of these beliefs may be motivating and some non-motivating in the process of preparation. Table 5 summarizes the salient beliefs and their probable implications.

**Table 5. Behavioral, normative and control beliefs and their probable motivating and non-motivating implications for the earthquake preparedness:** 

	Motivating	Non-motivating
Behavioral beliefs	It reduces the casualties and damages	It causes anxiety
	It helps people not be confused	It is useless
	It manages families until the relief workers arrive	
	It helps to organize families after the disaster	
Normative beliefs	The family and friends approve it	The family and friends are indifferent to it
		Some of the family members and friends disapprove it
Control beliefs	Training facilitates it	Daily involvements are obstacles
	There is no obstacles	Indolence and nonchalance are obstacles
		Financial constraints make it difficult
		It is not destined by human’s will
		Living in an apartment makes it difficult
		The family preparedness is dependent on the community preparedness

## **Discussion**

Few studies have tried to identify the belief-behavior relationships in the earthquake preparedness. In the study by Becker and his colleagues^58^, “the positive outcome expectancy” restated as the general phrase “preparing helps me in a disaster" is similar to the belief in our investigation: “the earthquake preparedness is useful for my family”. Its general form may show that the positive expectations are not that clear to the people. To state the matter differently, some people are doubtful about the benefits of earthquake preparedness, because of their beliefs about the earthquake hazard and their scant knowledge, mainly via mass media, about Tehran’s structural vulnerability. It means that the behavioral belief of “uselessness of preparation” is possibly formed by their exaggerated vulnerability assessment of the town structures and the false beliefs about the hazard. However, it is important to distinguish between the “usefulness” of preparedness in general and its “helpfulness” in particular. It seems that the interviewees who believed that preparedness was useful, but doubted about its helpfulness in Tehran, detected such a difference. At the present stage, this is only a speculation that needs further investigation. Moreover, emotions, feelings and social norms are likely to influence shaping these beliefs^58^.

Taking the worst possible scenario, stated or implied in many of the interviews, may be associated with their unrealistic assessment of the earthquake and its consequences. Two beliefs seriously challenging the behavioral belief about the preparedness effectiveness are: 1) in earthquakes, all buildings collapse and 2) it is not possible to access preparedness kits when an earthquake happens. These beliefs have made some interviewees believe that “the earthquake preparedness in apartments is useless”. The assumption behind such a belief is that earthquake destroys all of the apartments. In the previous studies, the implications of the house ownership for preparedness have been mentioned^59^, while the effect of house type on preparation is probably a new finding not included in those studies.

The behavioral belief that “actions for the earthquake preparedness can create anxiety among family members” is apparently a new finding too. In the previous studies, the source of anxiety was the hazard itself, not the hazard preparedness^12^^,^ 60.

The lack of information about family members’ views shows the absence of any talks about the preparedness in the families. Some of the interviewees expressed this issue explicitly. "Not talking" about the earthquake preparedness in the families could be considered as "indifference". This normative belief can be related to the technical term "critical awareness"^61^ which in this particular case is the amount of thinking or talking about earthquakes and its risks^12^.

In the previous studies in Tehran, public education, especially the role of television in informing, was highlighted^62^. This emphasis indicates that the people believed that their lack of preparedness is a result of their lack of knowledge. This control belief is also considered by some people from other countries^63^. It seems that the people’s emphasis on educating specially via television programs can be interpreted as shifting responsibility towards formal institutions. In other words, although “the public education via television facilitates getting prepared for people" may be a true belief, it would show that how people justify themselves not being prepared^12^. In addition, too much emphasis on informing and educating can be misleading, since it may be thought that people who know how to reduce the risk and its consequences inevitably prepare themselves. This assumption was not supported by several studies^14^^,^ 36^,^ 64 . On the contrary, informing and educating may sometimes cause a decrease in the perception of risk and preparedness level^12^ or at least they would not have a significant effect on the risk perception^6^. The control belief “the daily involvements are obstacles for preparedness” is often regarded as “lack of time”. Tehran inhabitants, like the people from some other countries^65^ , considered this factor as a barrier to preparedness. Lack of time for a particular thing usually means that thing is not of priority. This is clearly expressed by some of the interviewees in this study. This belief may be formed due to the people’s poor perception of risk. As in the previous studies, including a study conducted in Tehran^6^ showed that those with a greater perception of risk were more prepared. “Indolence and nonchalance” might be associated with the poor perception of risk. On the other hand, this belief may show the role of personality characteristics in the disaster preparedness. More researches are needed to be conducted on this issue.

Financial constraints usually expressed as “not having enough money”. It is a control belief emphasized by the people in similar researches^63^. The control belief “I have not enough money, so I cannot prepare my family” also shifts the responsibility from the families towards the government. This finding is consistent with the findings of the previous researches^11^^,^ 14.

Fatalism which almost in all of the cases was rephrased as the religious belief in “destiny” implies the concept that disaster and its consequences are “being out of control”. Even those considering the earthquake preparedness as necessary, accepted the role of an element not controlled by the willpower of human. Some of the studies have shown that fatalism can reduce the level of earthquake preparedness^22^. Although fatalism has been seen more in people with lower incomes^63^, it seems this control belief relates to the religious beliefs, whatever they would be, more than any other factors^67^.

## **Limitation**

The main limitation of the study is that, even though data saturation was reached, since these beliefs are expressed by those who are not prepared for the earthquake, the researchers cannot fully make sure that the same results would be yielded among the more prepared groups. Therefore, this study needs to be replicated to see whether the current results are found elsewhere. If so, a follow-up quantitative study is needed to explore the actual percentage of the people holding these beliefs. Another limitation was sampling procedures used in this research. ****A purposive sample is not representative, although a maximum variation sample aims to be in certain situations, like in this study, more representative than a random sample.

## **Conclusion**

In the present study, the salient behavioral, normative and control beliefs of Tehran inhabitants about the earthquake preparedness are defined and discussed based on TPB. The fidings are indicative of the fact that the Tehranis’ preparedness behaviors can be influenced by these beliefs. Recognition of these beliefs may assist policy makers and executives to develop a better understanding of the origins of the attitudes, the subjective norms, and the perceived barriers to the preparedness. In the other words, with such an understanding, they may determine the factors that influence the public preparedness behaviors. Any interventions to change these behaviors should be made based on this knowledge.

## Corresponding Author

Mehdi Najafi is the corresponding author and can be contacted via najafirc@gmail.com.

## **Competing Interest**

The authors have declared that no competing interests exist.

## Source of funding

The authors received no specific funding for this work.

## **Data Availability**

All relevant data are reported in the paper.
